# No artificial intelligence authors, for now

**DOI:** 10.1016/j.patter.2023.100731

**Published:** 2023-04-14

**Authors:** Andrew L. Hufton

**Affiliations:** Editor-in-Chief, *Patterns*

In February, Elsevier updated its authorship policies to provide guidance on the use of artificial intelligence (AI) tools during manuscript writing. Authors are now asked to acknowledge their use and are reminded that they must bear ultimate responsibility for the accuracy and appropriateness of the text in their paper. These policies also clarify, importantly, that AI tools may not be listed as authors. This applies to all Cell Press journals, including *Patterns*. Other publishers and journals have made similar statements banning AI tools from being listed as authors (see e.g., Thorp, Science *379*, 313 or Editorial, Nature *613*, 612).

This is not intended to limit how our authors use machine learning or AI methods in the course of their research. When such techniques are used in a study, or are the focus of the paper itself, they should be transparently described in the paper’s methods section and should be supported by open source code, model files, and training and testing datasets.

These new policies were prompted by a number of papers appearing in journals and on preprint servers over the last several months that listed as an author ChatGPT, an advanced conversational AI agent developed by OpenAI that is capable of writing plausible scientific text, holding lengthy conversations, and even of generating functional computer code. *Patterns* has already published a series of short opinion pieces this year exploring its capabilities and discussing its potential consequences for education and potential abuse as an aid to scientific fraud (see also Stokel-Walker, Nature *613*, 620–621; Sample, Guardian, January 26, 2023).

It is hard to escape the feeling that these new policies, while sensible, may be ephemeral—a product of this very particular point in history where AIs are advanced enough to write complex scientific text but not advanced enough for us to recognize in them the intangible qualities that may indicate a moral right to authorship. At present, AI tools lack the agency and independence needed to take *responsibility* for content they produce or to provide *consent* for publication—two qualities that are central to our scholarly concepts of authorship. Nonetheless, it is not hard to imagine that AI agents might eventually satisfy these criteria. Indeed, science fiction has been anticipating this development for some time.

With massive monetary investments pushing ever faster development of ever more human-like AI agents, it seems likely that we will be back to discuss this topic further. It also seems inevitable that any discussions of “rights” for human-like AI agents will be fraught and controversial, given that the commercial interests driving investment are often looking to use AI tools specifically to replace human writers.

Setting aside these more economic issues, it seems reasonable to posit that these conversational AI tools fascinate us in part because they provide a fractured-mirror view of our own humanity. Like Narcissus seeing his reflection for the first time, it is hard to look away. When we discuss the potential for AIs to have rights, including authorship rights, are we not therefore, at some level, discussing our own rights? Given that the protection of basic rights for humans is, optimistically, a work in progress, this is something, in principle, we should all welcome. At the same time, taking a warning from Narcissus, we should not become so entranced by this reflection that we lose sight of truly important matters. For now, these issues remain theoretical and far removed from the kinds of concrete human rights debates that have real impacts on individuals’ lives.

So, for the time being, please do not list AI tools in the author list. *Patterns* will, however, keep an open mind with regard to the future. If and when AI agents are able to satisfy more of the criteria outlined above, we will revisit our stance.

